# Supplementary figures and images for: Association of Reallocating Time in Different Intensities of Physical Activity with Weight Status Changes among Normal-Weight Chinese Children: A National Prospective Study
Source: Int J Environ Res Public Health. 2020 Aug 10;17(16):5761. doi: 10.3390/ijerph17165761 (PMC7459607; doi:10.3390/ijerph17165761)

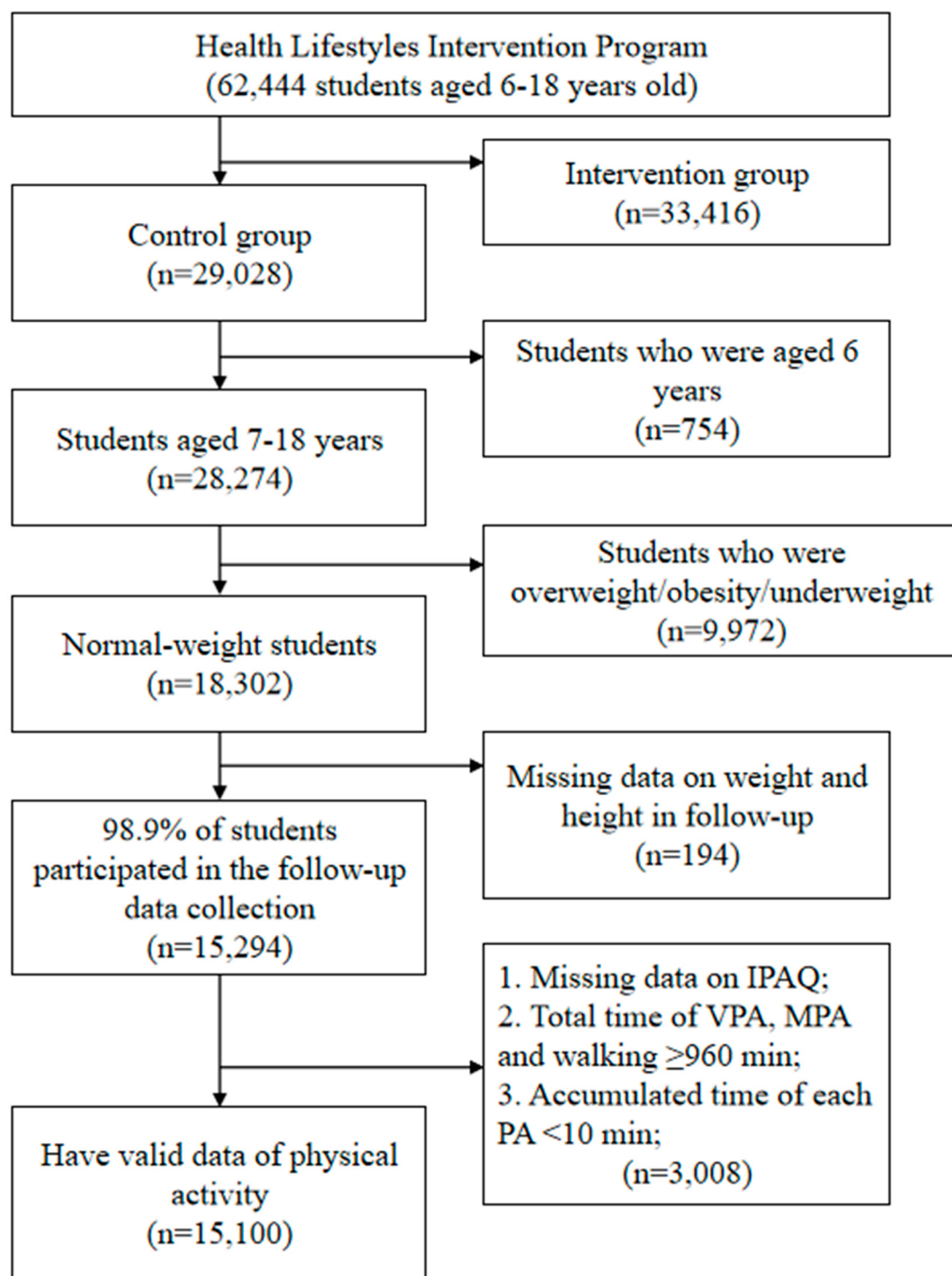

**Figure S1** Participants flow diagram

Supplement: Supplementary file 1 [file ijerph-17-05761-s001.pdf]
